# Access granted! barriers endure: determinants of difficulties accessing specialist care when required in Ontario, Canada

**DOI:** 10.1186/1472-6963-13-146

**Published:** 2013-04-22

**Authors:** Daniel W Harrington, Kathi Wilson, Mark Rosenberg, Scott Bell

**Affiliations:** 1 Department of Geography, University of Toronto Mississauga, 3359 Mississauga Rd. N., W.G. Davis Bldg., Mississauga, Ontario, L5L 1C6, Canada; 2 Department of Geography, Queens University, Kingston, Ontario, Canada; 3 Department of Geography and Planning, University of Saskatchewan, Saskatoon, Saskatchewan, Canada

**Keywords:** Access to health care, Specialist care, Difficulties

## Abstract

**Background:**

In the Canadian context, health care services are governed by the Canada Health Act, which ensures that primary care doctors, specialists, hospitals and dental surgeries are covered through provincial health insurance plans. This ensures access to medically necessary health care services for all Canadians regardless of ability to pay. Despite this important piece of legislation, research has shown persistent inequalities in access between and within socio-demographic groups, and geographic areas. To date, most research has focused on access to primary care, with much less attention paid to specialist care as an important component of the health care continuum. Thus, the objectives of this research are to address this gap in knowledge by examining the factors associated with difficulty accessing specialist services, and the reasons why particular subpopulation groups report experiencing difficulties.

**Methods:**

This research uses multivariate logistic regression to analyze data from the Canadian Community Health Surveys’ optional content from the province of Ontario (n=21,526) related to accessing specialist health care services. The multivariate logistic regression model identifies several subpopulation groups that are more likely to report difficulty accessing specialist care when required. Cross-tabulations are subsequently used to establish the main reasons why difficulties are faced.

**Results:**

Over 26% of respondents required a specialist visit in the 12 months preceding administration of the survey. Of these, 22% reported difficulty accessing specialist care. Those with difficulties were more likely to be immigrants, post-secondary educated, and have one or more chronic conditions. People living in urban health regions were also more likely to report difficulties accessing care. Primarily wait times were cited as reasons for these difficulties, followed by a perceived lack of availability.

**Conclusions:**

There are difficulties faced by the general population as a whole (e.g., wait times) as well as particular difficulties experienced more frequently by certain groups (e.g., transportation, language, and cost barriers for newcomers). These issues are important, as they may discourage individuals from using necessary health care services, and may contribute to feelings of dissatisfaction with the health care system.

## Background

Health care services in Canada are governed by the Canada Health Act [CHA], which ensures that primary care doctors, specialists, hospitals, and dental surgeries are covered through provincial health insurance plans [1]. Thus the CHA mandates access to medically necessary health care services for all Canadians regardless of ability to pay. Despite the implementation of the CHA, research has shown persistent inequalities in access to health care between socioeconomic groups, and particular geographic areas (e.g., urban versus rural dwellers) [2]. As an important determinant of the health of populations, such inequalities and inequities in access highlight an important domain for research on health services.

At the international scale, recent data indicate that access to specialists in Canada is not as good as it is in other countries. For instance, 57% of Canadians wait at least four weeks for access to specialty care, second only to the United States (60%), and more than twice the proportions from countries that rank highest - Germany (23%) and New Zealand (22%) [3]. Further, 32% of Canadians wait one to three months for access to specialty care, with 11% waiting longer than that [4].

According to Aday and Andersen [5], the determinants of health care access and use are generally categorized as predisposing, enabling, and need-based factors. Predisposing characteristics such as age, sex, and other demographic and social factors represent the likelihood that an individual will seek out and use health services. Enabling factors are those that enable or inhibit the use of health services. These may exist at the individual level (e.g., level of income or education, access to a personal vehicle) and at the community level (e.g., physical availability of health services). Finally, need factors refer to actual health status of an individual (e.g., presence of chronic conditions, self-rated health); these are often associated most strongly with the use of health services [5].

Studies of specialty care have, in general, considered a range of predisposing, enabling, and need factors that exist at patient, provider, and community-levels as determinants of access and use; there has also been a distinct focus on the role of socioeconomic status (SES) as measured by income or education. For example, [6] found that after controlling for need characteristics, lower income individuals and those with lower levels of education were less likely to visit specialists than those with relatively higher SES. Similarly, in an international analysis of five countries, when needed compared to those with above-average incomes Canadians with below-average income were found to be significantly more likely to find it ‘extremely’ or ‘very’ difficult to see a specialist [7]. The inequality with respect to income has been echoed by a recent analysis of general practitioner and specialist services across nine European countries [8].

At the community-level, in terms of availability of specialist services, it has been clearly shown that living in a large urban area may enable use of specialty care. For example, in the largest urban centres in Canada there are 11.0 specialists per 10,000 populations, compared to 1.0 per 10,000 in rural areas [9]. Potentially related to the relative absence of these services, rural dwellers in Canada [9,10], and internationally [11-13] are also less likely to use specialist services than those living in urban areas.

Beyond studies of utilization of specialist services, the majority of research has focused on understanding the factors that determine referrals to specialists by primary care physicians (PCPs) [10,14-21]. The process of referral from PCP or other health professional to specialist is a key coupling in the health care continuum. That is, while patients can self-refer to primary care services, access to specialty care is typically dependent upon a referral [6]. However, the referral process is not straightforward, and is recognized as being a complex process dependent upon the patient (e.g., age, gender, socioeconomic status, disease stage), the practitioner (e.g., age, gender), and community-level factors (e.g., urban/rural, presence of a medical school, specialist supply) [10,15]. The process of referral to secondary specialist care has important implications for patient health as well as efficiency of health care systems, and efforts are being made to improve the current process in Canada, and elsewhere through interventions and iterative development of best practices [3,22].

Though there are important inequalities that have been identified throughout the process of accessing specialty care it is important to consider the potential barriers that exist following a referral. That is, who among individuals who require specialist care face difficulties related to access? The aim of this study is to examine difficulties accessing specialist care in Ontario, Canada, and explore the determinants of experiencing such difficulties.

## Methods

The Canadian Community Health Survey [CCHS] is an annually collected cross-sectional telephone survey focused on gathering information about health status, health behaviours, and health care utilization from the Canadian populace over 12 years of age. The CCHS public use microdata file is available free of charge directly from Statistics Canada, or through subscribed Canadian postsecondary institutions through the Data Liberation Initiative. This study is based on analysis of data specific to access to specialist health care services from the province of Ontario’s optional content module from the 2010 CCHS public use microdata file [23]. In addition to common survey content which is asked of all survey respondents, health regions and/or provinces may choose to have optional content collected that may resonate with provincial or regional priorities. Only the province of Ontario opted to have data on access to specialist services collected for the 2010 cycle of the CCHS [24]. The sample size for Ontario is 21,536 (response rate = 70.0%). It should be noted that Aboriginal people living on-reserve, full-time members of the Canadian Forces, and the institutionalized population are excluded from the CCHS sampling frame by design [23].

### Study variables

#### Dependent variables

Three dimensions of access to specialist services were central to this analysis. These outcomes are related to requiring a specialist visit in the previous year, difficulty accessing specialist services, and types of difficulties faced. These variables were based on the following questions in the Ontario optional content (2010 CCHS question code in parentheses):

1. In the past 12 months, did you require a visit to a medical specialist for a diagnosis or a consultation? (ACC_10)

2. In the past 12 months, did you ever experience any difficulties getting the specialist care you needed for a diagnosis or consultation? (ACC_11)

3. What type of difficulties did you experience? Mark all that apply. (ACC_12A through ACC_12M)

a. Difficulty getting a referral

b. Difficulty getting an appointment

c. No specialists in the area

d. Waited too long – between booking and appointment

e. Waited too long – to see the doctor (i.e. In-office waiting)

f. Transportation – problems

g. Language – problems

h. Cost

i. Personal or family responsibilities

j. General deterioration of health

k. Appointment cancelled or deferred by specialist

l. Unable to leave the house because of a health problem

m. Other

Due to small numbers and missing values for some of the types of difficulties, the above 13 types were recorded into five broad categories for analysis as follows: (i) Availability (3a, 3b, 3c, 3 k); (ii) Wait times (3d, 3e); (iii) Transportation/Language/Cost (3f, 3 g, 3 h); (iv) Health related (3j, 3 l) and (v) Personal/Other (3i, 3 m).

#### Independent variables

Known determinants of health care access per Aday and Andersen’s [5] behavioural model for access to medical care related to predisposing characteristics, enabling characteristics, and need were included as potential covariates of difficulty accessing specialist care. All variables were coded as they appear in Table 1. The reference categories for most variables were selected to be the category least likely to be associated with having difficulty accessing specialist care in the previous 12 months (outcome variable). Marital status, age, sex and time since immigration represent predisposing characteristics. Marital status was coded as living with a partner (legally married and common-law married) versus not (divorced, separated, widowed, never married). Age was categorized into four levels; under 30 years of age, 30 to 44, 45 to 59, and 60 and older. Time since immigration was coded as being born in Canada, less than 10 years of living in Canada, and 10 years or more of living in Canada.

**Table 1:** Predisposing, enabling and need determinants of access

**Determinant type**	**Variable**	**Coding**
Predisposing Factors	Sex	Female (Reference) / Male
	Age	Under 30 (Ref.) / 30–44 / 45–59 / 60+
	Marital Status	No partner (Ref.) / Living with a partner
	Time since immigration	Born in Canada (Ref.) / Less than 10 years / More than 10 years
Enabling Factors	Education	Less than high school (Ref.) / High school / Post-secondary educated
	Income	Not low income (Ref.) / Low income
	Family Doctor	No (Ref.) / Yes
	Health Region	City of Toronto (Ref.) / Urban / Rural
Need Factors	Chronic conditions	No chronic conditions (Ref.) / 1–3 conditions / 4+ conditions

Enabling characteristics included income, education, having a family doctor, and health region of residence. Low income in this study was defined by having a household income – adjusted by household and community size to – in the province of Ontario’s lowest quintile. Highest level of education was recoded to less than secondary school, secondary school only, and post-secondary education (e.g. University education, College education and higher). Statistics Canada uses the 35 health regions in Ontario as the primary sampling frame for the CCHS, and these were conceptualized as being enabling characteristics. Health regions were grouped into three categories: ‘Urban,’ ‘Rural,’ and the ‘City of Toronto.’ The City of Toronto was separated from the other urban health regions because it is the largest metropolitan centre in Ontario, and the main centre for secondary and tertiary health care in the province. Urban areas were those that satisfied one of two criteria: (1) per guidelines proposed by the Organization for Economic Co-operation and Development [OECD] the health region had more than 150 people per km^2^[25], or (2) the health region contained a Census Metropolitan Area that represented at least 85% of the population of the region. Census Metropolitan Areas in Canada are municipalities, or clusters of municipalities around a central core, with a total population over 100,000 people of which 50,000 live in the central core [26]. There is no universal definition of ‘urban’ or ‘rural’ in the Canadian context at the health region level, thus the addition of the second criteria. Eighty-five percent of a region’s population living in a CMA was used as the cut-off point, as the most recent statistics show that 85% of the population of Ontario lives in urban areas versus rural areas [27]. Data used to calculate the proportion of the health region living in a CMA were available from the 2006 Canadian Census community profiles. The use and usefulness of this procedure for defining health regions as urban versus rural will be discussed further in the limitations section of the paper.

The CCHS collects data on a range of chronic conditions used to create the variable measuring health need. These chronic conditions include: asthma, fibromyalgia, arthritis, back problems, high blood pressure, migraine headaches, chronic obstructed pulmonary disorder (COPD), diabetes, heart disease, cancer, stomach or intestinal ulcers, effects of a stroke, urinary incontinence, bowel disorders, chronic fatigue syndrome, multiple chemical sensitivities, mood disorders, and anxiety disorders. A total number of chronic conditions were tallied for each respondent, and coded as ‘No chronic conditions,’ ‘1-3 chronic conditions,’ and ‘4 or more chronic conditions.’

### Analysis

All univariate and multivariate analyses are weighted to population weights provided by Statistics Canada in the public use microdata file for the 2010 CCHS. These weights, calculated based on Census information, ensure that the sample is representative of the population of Ontario. Regression analyses are weighted by a probability weight derived from the population weights. A multivariate logistic regression model was used to explore the determinants of reported difficulty accessing specialist care when needed in the prior year. Coefficients are presented in the results below as odds ratios (ORs) with 95% confidence intervals (CIs). Odds ratios can be interpreted as the odds that a respondent experienced difficulty accessing specialist care relative to the reference category of the variable, adjusted for all other variables in the model. An odds ratio greater than 1 would indicate that a respondent with a particular characteristic was more likely to have difficulty, while an odds ratio less than one indicates that a respondent would be less likely. Subsequent bivariate analyses of type of difficulty experienced were conducted for several sample sub-groups using crosstabulation evaluated with the chi-square statistic. All analyses were performed using R v. 2.15.1.

## Results

Table 2 presents descriptive information of the entire study cohort, compared to sub-samples of the cohort that required a visit to a specialist, stratified by those who did (n = 1143), and did not (n = 4633) experience difficulties accessing care. These descriptive statistics have all been weighted to the total Ontario population, and are presented with 95% confidence intervals generated using a bootstrap technique. This table gives an indication of how those who required specialist care in the previous year compare to the overall general CCHS Ontario sample.

**Table 2:** Sample description with 95% confidence intervals

		**Required a specialist in previous 12 months**	
**Attribute**	**CCHS Ontario (%)**	**Experienced difficulty (%)**	**Did not experience difficulty (%)**
Female	51.0 ± 1.2	57.3 ± 6.1	56.8 ± 7.0
Under 30	28.0 ± 1.1	13.9 ± 3.3	16.5 ± 1.7
30-44	24.2 ± 1.2	25.9 ± 4.9	21.6 ± 2.6
45-59	26.3 ± 1.2	38.0 ± 6.3	28.4 ± 2.6
60+	21.6 ± 0.8	22.2 ± 3.8	33.5 ± 2.1
Living With Partner	59.0 ± 1.3	70.1 ± 4.5	64.6 ± 2.4
Canadian Born	67.2 ± 1.3	60.7 ± 6.1	70.4 ± 2.7
Immigrant: < 10 Years	8.2 ± 0.8	10.7 ± 4.5	6.1 ± 1.8
Immigrant: 10+ Years	24.6 ± 1.2	28.7 ± 5.7	23.5 ± 2.4
Less Than $20,000	7.5 ± 0.7	9.1 ± 3.4	8.7 ± 1.6
$20,000-$39,999	15.6 ± 0.8	14.3 ± 3.8	16.2 ± 1.8
$40,000-$59,999	16.9 ± 1.0	15.2 ± 4.2	14.9 ± 1.7
$60,000-$79,999	16.3 ± 1.2	13.9 ± 4.4	14.5 ± 1.8
$80,000 And More	43.7 ± 1.4	47.6 ± 6.5	45.8 ± 2.9
Less Than Secondary School	19.8 ± 0.8	7.9 ± 2.3	14.4 ± 1.6
Secondary School	17.4 ± 1.0	11.9 ± 3.1	15.8 ± 1.8
Post-Secondary	62.7 ± 1.2	80.2 ± 3.7	69.8 ± 2.3
Has a Family Doctor	89.4 ± 0.9	93.1 ± 3	95.0 ± 1.0
No Chronic Conditions	54.5 ± 1.3	26.4 ± 4.9	37.8 ± 2.7
1-3 Conditions	41.1 ± 1.3	61.1 ± 5.5	54.1 ± 2.5
4+ Conditions	4.5 ± 0.4	12.5 ± 2.8	8.1 ± 1.1
City of Toronto	20.9 ± 1.2	14.8 ± 4.3	22.2 ± 2.5
Urban	56.6 ± 1.3	63.8 ± 5.2	54.5 ± 2.6
Rural	22.4 ± 0.8	21.3 ± 4.1	23.3 ± 1.7

With respect to access to specialists, 26.5% of the population in Ontario reported that they required a consultation with, or diagnosis from a specialist in the previous 12 months. Of those requiring a specialist visit, 22.0% reported that they experienced some difficulty getting the specialist care they needed.

After filtering the sample to include only those respondents who required a specialist visit in the previous year, a multivariate logistic regression model was used to predict the likelihood that a respondent experienced difficulty accessing required specialist care (Table 3). Predisposing, enabling, and need factors all emerged as important predictors of difficulty accessing care. In terms of predisposing characteristics, only a marginally statistically significant difference (i.e. p<0.10) between males and females was found, suggesting a slight trend towards males reporting more difficulty than females. Age was shown to have an effect, indicating that older respondents over the age of 60 are about half as likely as other age groups to report difficulties accessing specialist care. Time since immigration was highly significant in the model. As suggested by the adjusted ORs, immigrants in general were much more likely to report difficulties accessing specialist care in Ontario in comparison with the Canadian-born population. More specifically, newcomers to Canada (i.e., those living in the country for less than 10 years) were almost three times as likely to report experiencing difficulties accessing specialist care compared to their Canadian-born counterparts. More established immigrants (i.e., 10 years or more in Canada) were still more likely to experience difficulties than those who are Canadian-born, albeit less likely than newcomers [OR=1.72, 95% CI: (1.44, 2.07)]. Marital status was not significantly associated with difficulty accessing care.

**Table 3:** Multivariate logistic regression of difficulty accessing specialist care

**Factor**	**OR**	**95% CI**	**Significance**
Intercept	0.057	(0.035, 0.091)	***
*Sex (ref: Female)*			
Male	1.150	(0.987, 1.340)	.
*Age (ref: Under 30)*			
30-44	1.154	(0.888, 1.503)	
45-59	1.173	(0.908, 1.520)	
60+	0.489	(0.369, 0.648)	***
*Living arrangement (ref: no partner)*			
Partner	1.031	(0.863, 1.234)	
*Immigrant status (ref: Canadian born)*			
Less than 10 years	2.971	(2.226, 3.957)	***
10 or More years	1.724	(1.437, 2.067)	***
*Income*			
Low Income	0.991	(0.806, 1.214)	
*Education (ref: Less than high school)*			
High School	1.215	(0.876, 1.695)	
Post-sec. graduate	1.853	(1.415, 2.457)	***
*Family doctor (ref: No family doctor)*			
Has family doctor	0.786	(0.580, 1.075)	
*Chronic conditions (ref: None)*			
1-3 conditions	2.179	(1.827, 2.607)	***
4+ conditions	3.835	(2.867, 5.123)	***
*Urban/rural (ref: City of Toronto)*			
Urban Health Units	2.021	(1.626, 2.526)	***
Rural Health Regions	1.721	(1.323, 2.246)	***

Significance codes p<0.10=‘.’; p<0.05=‘*’; p<0.01=‘**’; p<0.001=‘***’.

Of the individual-level enabling characteristics, only education emerged as significant in this model. In particular, respondents who had completed some type of post-secondary education were more likely to report difficulties accessing specialist care in the previous 12 months [OR=1.85, 95% CI: (1.42, 2.46)]. Neither income nor having a family doctor was significantly associated with difficulty accessing specialist care in this model. At the regional level, it was clear that respondents living in the City of Toronto Health Unit were less likely to report difficulties in accessing specialist care compared to their counterparts living in other urban regions (OR=2.02, 95% CI: [1.63, 2.52]) or rural health units (OR=1.72, 95% CI: [1.32, 2.25]).

Finally, in terms of the variable measuring health need in this model, number of chronic conditions was associated with difficulty accessing care at a highly significant level. Specifically, as the number of chronic conditions increased from 1–3 conditions to 4 or more conditions, the adjusted odds of experiencing difficulty accessing specialist care increased from 2.18 [95% CI: (1.83, 2.61)] to 3.84 [95% CI: (2.87, 5.12)] compared to respondents with no reported chronic conditions.

### Type of difficulty experienced

**Figure 1:**
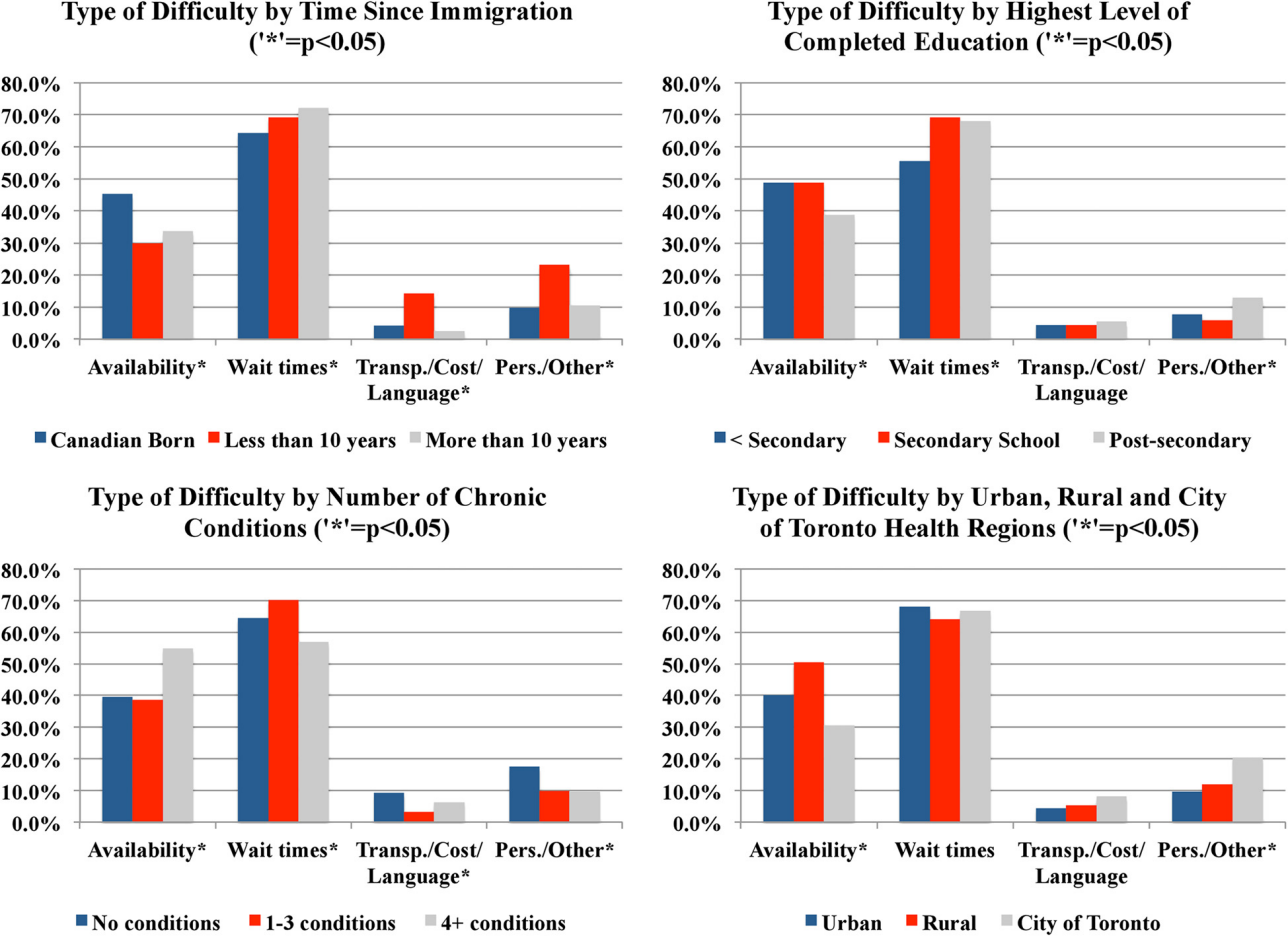
Type of difficulty experienced by four selected characteristics.

The multivariate logistic regression revealed four key variables associated with an increased likelihood of experiencing difficulty (or difficulties) accessing specialist care when required in the previous year. These variables are as follows: (i) time since immigration; (ii) highest level of completed education; (iii) number of chronic conditions; and (iv) health region type (e.g., urban, rural). As a subsequent step in the analyses of these data, crosstabulations were used to explore the types of difficulties experienced by these groups (see Figure 1, Table 4). Note that due to the low number of respondents who cited health related issues as barriers to access, these data cannot be reported in cross-tabular format per Statistics Canada’s policies on data disclosure related to minimum sample size [28].

**Table 4:** Cross-tabulations of type of difficulty with sample sub-groups (with 95% confidence intervals)

**Sub-group**	**Availability (%)**	**Wait times (%)**	**Transport/Cost/ Language (%)**	**Personal/ Other (%)**
*Time Since Immigration*				
Canadian born	45.2 ± 2.9	64.3 ± 2.8	4.2 ± 1.2	9.8 ± 1.7
Less than 10 years	30.0 ± 2.7	69.2 ± 2.7	14.2 ± 2.0	23.3 ± 2.5
More than 10 years	33.7 ± 2.7	72.1 ± 2.6	2.5 ± 0.9	10.5 ± 1.8
*Education*				
Less than secondary school	48.9 ± 2.9	55.6 ± 2.9	4.4 ± 1.2	7.8 ± 1.6
Secondary school	48.9 ± 2.9	69.1 ± 2.7	4.4 ± 1.2	5.9 ± 1.4
Post-secondary	38.7 ± 2.8	68.0 ± 2.7	5.5 ± 1.3	13.0 ± 1.9
*Chronic Conditions*				
No chronic conditions	39.6 ± 2.8	64.4 ± 2.8	9.2 ± 1.7	17.5 ± 2.2
1-3 conditions	38.5 ± 2.8	70.2 ± 2.7	3.3 ± 1.0	9.8 ± 1.7
4+ conditions	54.9 ± 2.9	56.9 ± 2.9	6.3 ± 1.4	9.7 ± 1.7
*Urban/rural*				
City of Toronto	30.6 ± 2.7	66.7 ± 2.7	8.2 ± 1.6	20.5 ± 2.3
Urban	40.0 ± 2.8	68.1 ± 2.7	4.4 ± 1.2	9.7 ± 1.7
Rural	50.4 ± 2.9	64.1 ± 2.8	5.3 ± 1.3	11.9 ± 1.9

Overall, for all four groups, respondents who had experienced difficulty accessing specialist care in the previous year most frequently cited wait times as a source of difficulty (67.1%), followed by availability (40.8%). It should be noted that wait times in this study may refer to time spent waiting in the specialist’s office (12.7%), time spent waiting to secure an appointment with a specialist (77.9%), or, a respondent may have identified both as difficulties experienced (9.4%). Other sources of difficulty, related to transportation/cost/language (5.2%), Health-related (1.6%) and Personal/Other reasons (11.8%), were mentioned relatively less. Note that these percentages do not add up to 100%, as respondents could provide more than one source of difficulty.

Referring to the bivariate analyses of types of difficulty experienced (see Figure 1) some interesting findings emerge. Related to time since immigration, for example, Canadian-born respondents more frequently reported difficulty with specialist availability (45.2%) than immigrants living in the country for more than 10 years (33.7%) or new Canadians (30.0%). However, both newcomers (69.2%) and longer-term immigrants (72.1%) were more likely to report difficulties with wait times compared to Canadian-born respondents (64.3%). A higher percentage of newcomers reported they had experienced difficulties related to transportation, cost, or language (14.2%). Finally, newcomers were also much more likely to report personal or family responsibilities and other factors as difficulties they had experienced (23.3%) compared to Canadian-born (9.8%) and longer term immigrant respondents (10.5%).

Respondents with a post-secondary education experienced difficulties with availability less frequently (38.7%) than those who had not completed secondary school (48.9%) and those for whom secondary school was the highest level of completed education (48.9%). A higher percentage of those who had completed secondary school (69.1%) or higher levels of education (68.0%) reported difficulties related to wait times than those without a secondary school education (55.6%). Personal/family responsibilities and other difficulties were also reported more frequently by respondents who possessed post-secondary educations (13.0%). No significant differences were found in terms of transportation/cost/language barriers to access or health-related difficulties by education level.

Health care need in terms of number of chronic conditions was significantly associated with type of difficulty reported. Respondents with 4 or more chronic conditions were more likely to list availability as a difficulty (54.9%) compared to those with fewer (38.5%) or no chronic conditions (39.6%). However, these respondents reported difficulties with wait times less frequently (56.9%) than others. Finally, respondents without chronic conditions were more likely than those with chronic conditions to list transportation/cost/language and personal or family responsibilities as sources of difficulty.

There were some regional differences evident in this analysis as well. In particular, these were related to availability of specialist services. Respondents living in the City of Toronto Health Unit were much less likely to report availability as a source of difficulty (30.6%) compared to those living in other urban health regions (40.0%). Respondents from rural health regions were most likely to list availability as a difficulty experienced when trying to access specialist care (50.4%). There were no statistically significant differences around wait times, transportation/cost/language, or health-related difficulties; however, a higher percentage of respondents from the City of Toronto listed personal responsibilities/other difficulties (20.5%) than those from rural (11.9%) or urban regions (9.7%).

## Discussion

The goal of this study was to examine the determinants of experiencing difficulty accessing specialist care in the Province of Ontario, when a consultation or diagnosis from a specialist was required in the previous year. Of the 26.5% of the Ontario population who required specialty care, 22.0% reported having some type of difficulty accessing that care. Though the measurement of difficulty differed slightly, this result echoes that from Schoen and Doty [7] international comparison of health care access, that approximately 16% of the Canadian population reported finding it extremely or very difficult to see a specialist when needed. In terms of the types of difficulties experienced, wait times were cited most frequently by followed by availability of services. Wait times and access to services are the preeminent concerns of Canadians with respect to accessing primary care in Canada [29,30]. This study provides evidence that the same is true of specialist care.

We have shown evidence that the likelihood of experiencing difficulty is not equally distributed among the population of Ontario, and that a range of predisposing, enabling, and need factors at individual and regional levels impact difficulty accessing specialty care. Firstly, those reporting difficulty were more likely to be younger (under age 60). This is interesting given that older people are much more likely to be accessing specialist care than younger people [10], and may be accessing multiple specialists as part of their complete health care. While other research has alternatively reported that older people face more barriers to access than others [31], our findings suggest that older adults are less likely to report experiencing difficulties than their younger counterparts. Our findings might reflect the differences between getting a first appointment (perhaps more likely among younger individuals surveyed) in contrast to getting repeat appointments (perhaps more likely among older individuals).

The relationship between immigration status and difficulty accessing specialty care exhibited a very clear trend indicating that immigrants to Canada are more likely to experience difficulties accessing care compared to the Canadian-born population. More specifically, when accounting for time since immigration (more or less than 10 years), it is apparent that though after 10 years, immigrants continue to experience difficulties accessing specialists, new Canadians are almost three times as likely to report difficulties than the Canadian-born population. This result resonates strongly with work by others on access to primary health care among immigrant populations. For example, Sanmartin and Ross [32] found that new Canadians were 2.5 times as likely as Canadian-born to report difficulties in accessing routine and immediate (emergency) health care. Such findings have been linked to difficulties in obtaining a family doctor upon arrival into Canada, something that has been identified as an important challenge for the newcomer population [33]. However, the analysis undertaken in this study adjusts for the presence of a family doctor, which controls for this potential effect.

Some light can be shed on these results if the focus is shifted towards the types of difficulties faced (Figure 1). Specifically, new Canadians were much more likely to report barriers to specialist care related to transportation, cost, and language (14.2%), as well as those related to personal or family responsibilities and other factors (23.3%). Difficulties related to cost of health services may occur as a result of a combination of three processes: (1) there is a waiting period of three months for new permanent residents before the provincial health insurance plan will provide coverage; (2) non-status migrants and refugee claimants are not covered by the provincial health insurance plan; and (3) extended services including for example, prescription drugs and psychotherapy, are not covered under the provincial health insurance plan. Any combination of these processes may present difficulties in accessing specialist care for this particular population. Specific to language, though some efforts are being made in areas with large immigrant populations (See [34]), for many health services in Ontario, language interpretation is not available [35]. Related to personal or family responsibilities – while we are unable to unpack this type of difficulty using the CCHS public use microdata – a number of sociocultural barriers including beliefs about health, and perceptions of the health care system may potentially be playing some role [35]. Given that immigrants represent a large and expanding part of the total population of Ontario, it will be important for future research to continue to tease out these relationships with the goal of developing interventions to improve access to specialist services for this group.

Given the known inequities in access related to socioeconomic status, and more specifically income, it is somewhat surprising that there was no statistically significant effect of income on likelihood of experiencing difficulty in this analysis. This is likely a result of having a universal, public health insurance system that eliminates any direct financial payment barriers in accessing specialist services [1]. However, highest level of completed education was a significant determinant of experiencing difficulty. Overall there was a trend suggesting that the more education a person had completed, the more likely they were to have experienced difficulty. However, this effect was only significant for the highest level of education (post-secondary graduates) (OR=1.85). While education has not received much focus as a socioeconomic covariate of access to health care, it has been found previously to be associated with self-reported problems accessing primary care [36]. When looking at the types of difficulties faced by level of education, there is not much evidence to support why these differences exist, except that those with post-secondary education were more likely than the other groups to list personal or family responsibilities. While this relationship is not currently well understood, we suggest that the increased likelihood of reporting difficulties may be linked to differences in expectations of the health care system as level of education increases [37].

In terms of the needs-based factor included in this analysis, number of chronic conditions emerged as a highly significant predictor. In particular, a positive stepwise relationship emerged whereby respondents with 1–3 conditions were 2.2 times as likely to experience difficulties compared to those with no conditions, and those with 4 or more conditions were almost four times as likely to have had difficulties. As mentioned previously, needs-based factors are important to consider in all investigations of access to health care, as they are often most strongly related with related outcomes [5]. The findings reported here highlight an important inequality related to the population of Ontario who are the ‘sickest,’ and likely accessing care more frequently. What is particularly interesting is that those with four or more chronic conditions reported wait times as a difficulty less frequently than the other groups. However, they were more likely to report difficulties related to availability (i.e., related to getting an appointment, physical availability of services, and/or cancelation of appointments by the specialist).

Finally, there was a regional pattern of difficulty based on the respondents’ health region of residence. After adjusting for other variables in the model, respondents from the City of Toronto were found to be the least likely respondents to report having difficulty accessing specialist care. As mentioned previously, Toronto is the main centre of secondary and tertiary care in Ontario, so it makes sense that residents of Toronto have less difficulty accessing care. Interestingly, those from urban health regions were most likely to report difficulties accessing specialist care (OR=2.0) followed by those from rural regions (OR=1.7). This is counterintuitive because rural residents often have less accessible health care [6], due to barriers related to distance to care. In the analysis of type of difficulty experienced, indeed, a higher percentage of rural respondents experienced difficulties related to availability compared to urban respondents or Torontonians. Again, we surmise that expectations of access for rural residents may be much different than those from urban regions as an explanation for the differences in likelihood of experiencing difficulty. This echoes with the work of [37] who have reported that rural individuals are much less likely to report their waiting times as unacceptable, independent of the length of time they spent waiting. Rural residents may expect certain difficulties to be associated with their access of the health care system, and therefore have a higher tolerance than those from urban regions.

Some important questions arise as a result of the limitations of this study. The first of these relates to the types of specialty care being accessed by each individual. Potentially, the difficulties in accessing care may be heterogeneous among the specialties, and may differ by individual based on predisposing, enabling, and need factors. These data are not available in the CCHS but it remains an important area of future research. Secondly, measures of accessibility are based on self-reported access issues, and expectations may differ among different demographic, socioeconomic, and geographical sub-groups of the population. For example, there is no way to determine in CCHS whether two individuals who noted waiting times as a barrier actually waited the same amount of time, unless the sample is reduced to those who require a specialist visit for consultation or diagnosis of a new condition [23]. Further study focused on unpacking these expectations, including those using qualitative methodologies, will be crucial for developing deeper understandings of the relationships with difficulty accessing care reported in this study. Third, it is important to consider if the places in which people indicate access issues are indeed the places where the supply of specialists are problematic. That is, these data measure the ‘demand’ side of the access equation, and are presented here independent of the ‘supply’ side. Finally, the definitions used in this study to define health regions as urban or rural are not universally accepted methodologies in the Canadian context. Various competing definitions of urban and rural exist which raises the question of whether some of the results are artifacts of the definitions of urban and rural chosen. While we are confident that this is not the case, it would take a separate study to rule out any bias due to the definitions chosen.

## Conclusion

Despite the limitations addressed in the previous section, the study makes some important contributions to the literature on access to health care. Specifically, this study has focused on access to specialty care, which has received relatively less attention than access to primary care in the broader health care literature. In particular, by focusing on difficulties accessing specialty care when required in the previous year, this study provides an understanding of inequalities that exist beyond direct physical access and likelihood of referral to specialists. Further, the study provides some understanding of the types of difficulties faced by the general population (e.g., wait times), as well as particular difficulties experienced more frequently by certain segments of the population (e.g., transportation, language, and cost barriers for new Canadians; availability for rural residents). Understanding these issues is an important goal for researchers and policymakers, as direct and indirect barriers can potentially have deleterious health effects by discouraging individuals from using necessary health care services, and contribute to dissatisfaction with a health care system. In doing so, such knowledge can be mobilized towards improving access to specialist care.

## Competing interests

The authors declare that they have no competing interest.

## Authors’ contributions

All authors conceived this study, and discussed the results and implications of the study findings throughout all stages of the manuscript. DWH performed all analysis of the CCHS data. DWH and KW interpreted the data, and DWH wrote the initial draft of the paper. All authors read and approved the final manuscript.

## Pre-publication history

The pre-publication history for this paper can be accessed here:

http://www.biomedcentral.com/1472-6963/13/146/prepub
